# Investigating the relationship between diet quality, lifestyle and healthy eating index with severity and migraine attacks: a cross-sectional study

**DOI:** 10.3389/fnut.2024.1510809

**Published:** 2024-12-04

**Authors:** Marziye Feyzpour, Fatemeh Maleki Sedgi, Ghazal Baghdadi, Reza Mohammadifard, Mehran Rahimlou

**Affiliations:** ^1^Department of Nutrition, School of Public Health, Zanjan University of Medical Sciences, Zanjan, Iran; ^2^Food and Beverages Safety Research Center, Urmia University of Medical Sciences, Urmia, Iran; ^3^Department of Exercise Physiology, Faculty of Sports and Health Sciences, Tehran University, Tehran, Iran

**Keywords:** migraine, diet quality, healthy eating index, lifestyle factors, migraine severity

## Abstract

**Background:**

Migraine is a disabling neurovascular disorder often associated with comorbidities such as mental health disorders, cardiovascular diseases, and metabolic syndromes. While certain dietary triggers have been identified, the impact of overall diet quality on migraine severity and frequency is not well understood. This study aimed to evaluate the association between diet quality, lifestyle factors, and the Healthy Eating Index (HEI) with migraine severity and frequency.

**Methods:**

A cross-sectional study was conducted on 280 patients aged 18–50 years newly diagnosed with migraines. Dietary intake was assessed using a 147-item Food Frequency Questionnaire (FFQ), and diet quality was evaluated using the Lifelines Diet Score (LLDS) and HEI. Migraine-related disability and severity were assessed using the Migraine Disability Assessment (MIDAS) questionnaire and the Visual Analogue Scale (VAS), respectively. Logistic regression models were applied to examine the association between diet quality and migraine outcomes.

**Results:**

Higher LLDS and HEI scores were significantly associated with reduced odds of migraine-related disability. Participants in the highest LLDS tertile had an odds ratio (OR) of 0.68 (95% CI: 0.42–0.96; *p* = 0.02) for migraine disability. Similarly, the highest HEI tertile was associated with an OR of 0.58 (95% CI: 0.41–0.88; *p* = 0.025). For pain intensity, the highest tertile of LLDS showed an OR of 0.55 (95% CI: 0.38–0.75; *p* = 0.026), while the HEI showed an OR of 0.62 (95% CI: 0.45–0.85; *p* = 0.03).

**Conclusion:**

Higher diet quality, as measured by LLDS and HEI scores, is inversely associated with migraine severity and frequency. These findings suggest that dietary improvements may be a viable strategy for managing migraine symptoms.

## Introduction

Migraine is a debilitating neurovascular condition characterized by severe headaches, often accompanied by photophobia, phonophobia, nausea, vomiting, and heightened sensitivity to movement (1). It is frequently associated with mental health conditions, including depression and anxiety, sleep disturbances, chronic fatigue, and cardiovascular risk factors such as hypertension, diabetes, hyperlipidemia, and obesity (2–4). It is among the most common neurological disorders, with an estimated 14–15% of headache sufferers receiving a diagnosis of migraine (5). A recent meta-analysis found that the prevalence of migraine in the general population of Iran is 15.1% (6). The economic burden is significant; a systematic review reported annual healthcare costs ranging from £6,443 to £53,446 in some countries (7).

While the mechanisms underlying migraines remain unclear, environmental, hormonal, psychological, and dietary factors are potential contributors (8, 9). Certain foods, such as chocolate, caffeine, cheese, and alcoholic beverages, have been identified as common triggers (10–12). Dietary components may influence migraine pathophysiology through mechanisms involving neuropeptides, receptors, ion channels, inflammation, nitric oxide release, and vasodilation (13).

Although the evidence is limited, certain dietary interventions such as the ketogenic diet (14), elimination diets, and diets rich in anti-inflammatory foods show potential as effective approaches to managing migraines (15). However, individuals typically consume a variety of foods and nutrients simultaneously rather than in isolation, emphasizing the need to explore the combined effects, interactions, and cumulative impacts of diverse dietary components on migraines. Analytical methods, such as dietary pattern analysis and evaluations of overall diet quality, provide a more holistic understanding of the relationship between diet and migraines (13, 16).

Recent studies have examined the relationship between diet quality or dietary diversity and migraine attacks, finding that lower diet quality or diversity is associated with a higher frequency of attacks (17, 18).

However, to date, no studies have evaluated the combined effects of the LLDS and HEI on migraine severity and frequency. This study aims to address this gap by investigating the relationship between diet quality, lifestyle factors, and HEI with the severity and frequency of migraines.

## Methods

### Study setting

This cross-sectional study involved 280 patients, aged 18–50 years, who were newly diagnosed with migraines and referred to the neurology clinic at Vali-e-Asr Hospital in Zanjan, Iran, between March 2023 and July 2024.

### Study population and sample size

In this cross-sectional study, the population consisted of all patients aged 18 to 50 years who visited the neurology clinic at Vali-e-Asr Hospital in Zanjan. The sample size was calculated using G*Power software, referencing the study by Mirzababaei et al. (18). Based on the variability in migraine attack frequency, a statistical power of 80%, and a type I error rate of 5%, the required sample size was estimated to be 245 participants. To account for a potential 10% dropout rate, this number was adjusted to 265 participants. Ultimately, 280 individuals were enrolled to further increase the study’s power.

Eligibility criteria included being aged 18 to 50 years, attending the neurology clinic for the first time, not adhering to any specific diet, having a confirmed migraine diagnosis by a neurologist based on the International Classification of Headache Disorders (ICHD-3) criteria, and a willingness to participate. Exclusion criteria encompassed a history of kidney, liver, pancreatic, or cardiovascular diseases; diabetes; cancer; neurovascular or vasculitis disorders as reported by the patient or documented in medical records; malnutrition [Body mass index (BMI) < 18.5]; pregnancy; menopause; or refusal to complete the questionnaire. Participants with incomplete questionnaires, changes in treatment type, lack of cooperation, or implausible caloric intakes (above 4,000 kcal or below 600 kcal) were also excluded.

### Sampling method

**Figure 1:**
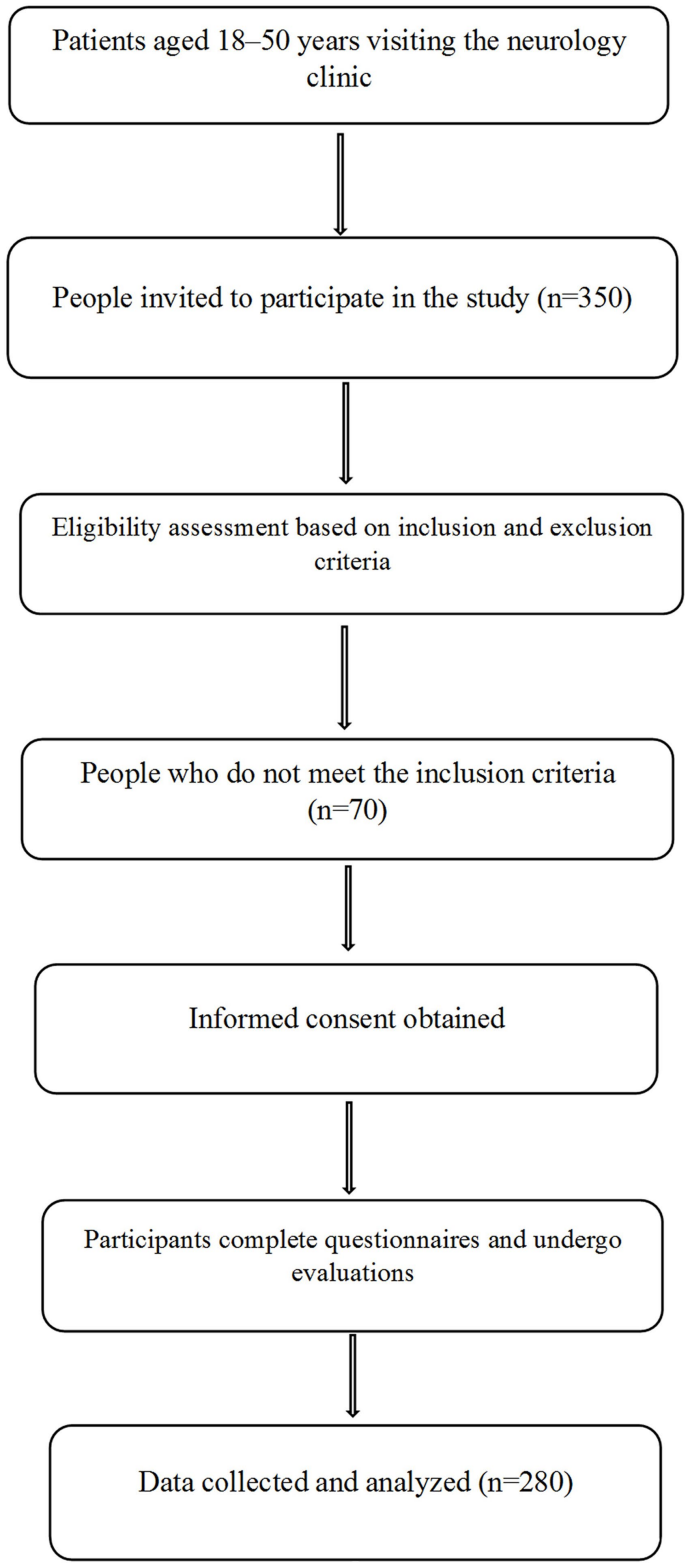
Flowchart of participant selection and study implementation.

A flowchart illustrating the study design, including participant eligibility, recruitment, and data collection processes, is provided in Figure 1. Participants were recruited using convenience sampling. Three researchers stationed at the neurology clinic of Vali-e-Asr Hospital identified patients recently diagnosed with migraines. Patients who met the inclusion criteria and agreed to participate were enrolled as study subjects. After a neurologist confirmed each patient’s migraine diagnosis based on the ICHD-3 criteria, patients were referred to the research team for further procedures. The study objectives were thoroughly explained, and informed consent was obtained from all participants. Eligible individuals, as defined by the inclusion and exclusion criteria, then underwent data collection, which included anthropometric measurements, a FFQ, a physical activity questionnaire, the MIDAS questionnaire, and the VAS.

The MIDAS questionnaire assessed the impact of migraines on participants’ daily lives over the past 3 months. It quantifies disability by measuring the number of days migraines interfered with work, household responsibilities, and social or leisure activities. MIDAS scores are categorized into four levels: little or no disability (score 0–5), mild disability (score 6–10), moderate disability (score 11–20), and severe disability (score 21 or higher). This tool provides a comprehensive evaluation of the burden of migraine-related disability, complementing the VAS’s assessment of pain intensity (19).

Pain intensity was measured using the VAS, a widely recognized tool for subjective pain evaluation. Participants rated their pain on a scale from 0 to 10, where 0 represented ‘no pain’ and 10 indicated ‘the worst possible pain.’ The VAS is straightforward to administer and provides a reliable, quantitative measure of pain intensity, suitable for tracking changes over time (20).

### Data collection tools

Food intake data were collected using a validated 147-item FFQ, which has been confirmed by previous studies (21, 22). Dietary intake analysis was conducted using N4 software, which converted all measurements into grams. All participants were newly diagnosed with headache conditions, and all questionnaires were completed by trained researchers. The FFQ assessed dietary intake over the past year, with participants recalling the frequency of food consumption (daily, weekly, or monthly).

FFQ data were analyzed to estimate dietary intake, with all measurements converted into grams using N4 software. Diet quality and HEI scores were calculated based on the FFQ data, using the USDA food composition table (23) to estimate energy and nutrient intake. Physical activity was recorded using the Iranian version of the International Physical Activity Questionnaire (IPAQ) (24), and anthropometric measurements were obtained from patient records.

The Pittsburgh Sleep Quality Index (PSQI) (25) was used to assess sleep quality among participants over the previous month. The PSQI is a validated self-reported questionnaire consisting of 19 items grouped into seven components: subjective sleep quality, sleep latency, sleep duration, habitual sleep efficiency, sleep disturbances, use of sleeping medication, and daytime dysfunction. Each component is scored from 0 to 3, with higher scores indicating poorer sleep quality. The total PSQI score, calculated by summing the component scores, ranges from 0 to 21, with a score above 5 indicating poor sleep quality. Trained researchers administered the PSQI and provided clarification to participants when necessary. A total score of 6 or higher indicates inadequate sleep quality.

The social status score was evaluated using a structured questionnaire designed to capture key socioeconomic factors, including educational attainment, occupational status, and income level. Each dimension was assigned a specific weight based on established scoring guidelines for social stratification. Educational attainment was categorized into three levels: primary education or lower (score = 1), secondary education (score = 2), and higher education (score = 3). Occupational status was classified as unemployed (score = 1), semi-skilled or skilled labor (score = 2), and professional or managerial roles (score = 3). Income was stratified into three tiers based on local economic benchmarks, with lower income (score = 1), middle income (score = 2), and higher income (score = 3). The final social status score was calculated by summing the scores from these three components, yielding a composite score ranging from 3 to 9.

### Dietary indices

In this study, diet quality was assessed using two primary indices: LLDS and HEI. The LLDS is a food-based dietary quality index that evaluates adherence to dietary guidelines by scoring the intake of specific food groups associated with positive health outcomes. LLDS includes the consumption of nine food groups: vegetables, fruits, whole grain products, legumes and nuts, fish, oils and soft margarines, unsweetened dairy, coffee, and tea, which have been shown to have positive effects on health, and three food groups: red and processed meat, butter and hard margarines, and sugar-sweetened beverages, which negatively affect health. Individuals’ food intake was expressed in grams per 1,000 kcal. For each food group, intake was divided into 1 to 5 quintiles, with 5 points awarded for the highest intake and 1 point for the lowest intake of positive food groups. For negative food groups, 5 points were awarded for the lowest intake and 1 point for the highest intake. The sum of the scores from the 12 components resulted in an LLDS score ranging from 12 to 60 (26, 27).

The HEI is calculated by assessing adherence to dietary recommendations across 13 components that represent various aspects of a balanced diet. These components include nine categories for food adequacy (e.g., total fruit, whole fruit, total vegetables, greens and beans, whole grains, dairy, total protein foods, seafood and plant proteins, and fatty acids) and four categories for foods to limit (e.g., refined grains, sodium, added sugars, and saturated fats). Each component is scored based on the proportion of intake that meets or exceeds the dietary recommendations. For adequacy components, higher intakes yield higher scores, while for moderation components, lower intakes of items like added sugars and saturated fats result in higher scores. Scores for each component range from 0 to a maximum component score (typically 5 or 10 points), and the sum of all component scores provides a total HEI score, which ranges from 0 to 100. Higher total HEI scores reflect closer adherence to dietary guidelines and, therefore, a higher-quality diet (28).

#### Data analysis method

Statistical analyses were performed using SPSS version 26 (IBM Corp., Armonk, NY, United States). Continuous variables were summarized as means ± standard deviations (SD) if they followed a normal distribution, or as medians and interquartile ranges (IQR) for non-normally distributed data. Categorical variables were presented as frequencies and percentages. The Kolmogorov–Smirnov test was used to assess the normality of continuous data.

Comparisons of baseline characteristics and dietary intake across tertiles of the indices (e.g., LLDS and HEI) were performed using appropriate statistical tests. For quantitative variables, one-way analysis of variance (ANOVA) was applied if the data were normally distributed. For non-normally distributed data, the Kruskal-Wallis test was used. For qualitative variables, chi-square tests or Fisher’s exact tests were employed, as appropriate. When significant differences were identified using ANOVA, Tukey’s *post-hoc* test was performed to determine pairwise differences between tertiles. To evaluate associations between diet quality indices and migraine outcomes, multivariate logistic regression models were used. Odds ratios (ORs) and 95% confidence intervals (CIs) were calculated for two primary outcomes: migraine-related disability, assessed by the MIDAS questionnaire, and migraine pain intensity, measured using the VAS. Three hierarchical models were constructed to control for potential confounders:

Model 1: Adjusted for total energy intake (kcal/day) and BMI. Model 2: Included additional adjustments for gender, social status score, sleep quality, and physical activity. Model 3: Further adjusted for daily water intake (glasses/day), salt consumption habits, and family history of migraines. Also, *p* < 0.05 was considered statistically significant for all analyses. All tests were two-sided, and results were reported with exact *p* where possible to enhance interpretability.

## Results

The mean age of participants was 35.19 ± 6.92 years, and the mean weight was 74.55 ± 16.52 kg. None of the participants were following a specific diet. As shown in Table 1, participants’ baseline characteristics and dietary intake varied across the tertiles of the LLDS and HEI. Additionally, the mean daily water intake among participants was 4.61 ± 2.69 glasses. Most participants (54.7%) were female, and 58.87% were married. Employment status varied, with 37.15% of participants being homemakers, 27.86% self-employed, and 34.99% either employed or students. About 49.65% of participants had never smoked, while 30% were current smokers. Physical activity levels were predominantly low, with 43.92% reporting low activity, 37.5% moderate, and 18.58% high. No significant differences in age, weight, BMI, or physical activity levels were found across the tertiles of the LLDS and HEI scores (*p* > 0.05). However, individuals in the highest tertile of both the LLDS and HEI reported significantly better sleep quality compared to those in the lowest tertile (*p* < 0.001).

**Table 1:** Overview of qualitative and quantitative variables in the study.

Variable (qualitative)		Frequency	Percentage
Gender	Female	153	54.7
	Male	127	45.3
Education level	High school diploma or lower	110	39.3
	Associate’s or Bachelor’s degree	130	46.4
	Master’s or Doctorate	40	14.3
Employment status	Homemaker	104	37.15
	Self-employed	78	27.86
	Employee or Student	98	34.99
Marital status	Married	164	58.87
	Single or divorced	116	29.3
Smoking status	Never smoked	139	49.65
	Current smoker	84	30
	Former smoker	57	20.35
Family history of migraine	No	173	67.8
	Yes	107	38.2
Medication use	Yes	129	46.1
	No	151	53.9
Preference for salty foods	Yes	103	36.78
	No	177	63.22
Adding salt during meals	Yes	163	58.20
	No	117	41.8
Physical activity level	Low	123	43.92
	Moderate	105	37.5
	High	52	18.58
Variable (quantitative)		Mean	SD^1^
Age (years)		35.19	6.92
Height (cm)		170.41	7.49
Weight (kg)		74.55	16.52
Waist circumference (cm)		89.48	14.15
Hip circumference (cm)		104.25	12.17
BMI		27.36	5.69
Waist-to-hip ratio		0.84	0.07
Daily water consumption (glasses)		4.61	2.69
Physical activity (MET/Min/day)		134.29	36.19
Social status score		5.91	2.87

1 Standard Deviation.

Table 2 presents the variations in baseline variables and dietary intake across the tertiles of the LLDS and HEI scores. The results show no significant differences in age, weight, BMI, or physical activity levels among the tertiles of LLDS and HEI scores (*p* > 0.05). However, individuals in the highest tertile of both LLDS (*p* < 0.001) and HEI (*p* < 0.001) reported significantly better sleep quality compared to those in the lowest tertile.

**Table 2:** Baseline quantitative variables and dietary intakes of individuals across LLDS and HEI score tertiles.

Variable	LLDS score tertiles				HEI score tertiles			
	T1 Mean ± SD	T2 Mean ± SD	T3 Mean ± SD	p*	T1 Mean ± SD	T2 Mean ± SD	T3 Mean ± SD	p*
Age (years)	34.28 ± 7.43	35.59 ± 8.34	34.40 ± 7.60	0.65	34.63 ± 8.49	35.22 ± 7.76	34.31 ± 8.15	0.71
Weight (kg)	76.18 ± 17.44	74.18 ± 16.37	74.56 ± 16.81	0.52	76.55 ± 17.83	75.19 ± 16.57	74.77 ± 16.47	0.63
BMI	27.65 ± 5.73	26.93 ± 4.77	27.18 ± 5.39	0.64	27.70 ± 5.82	26.69 ± 5.22	27.21 ± 5.31	0.59
Pittsburgh sleep quality index	6.83 ± 3.70	5.53 ± 3.35	4.32 ± 2.81	<0.001	6.95 ± 3.81	5.18 ± 3.19	4.25 ± 2.76	<0.001
Physical activity (MET/Min/day)	132.28 ± 35.73	136.22 ± 36.24	135.64 ± 33.45	0.78	129.25 ± 31.43	137.50 ± 35.72	134.83 ± 34.60	0.61

	T1 Mean ± SD	T2 Mean ± SD	T3 Mean ± SD	p*	p**	T1 Mean ± SD	T2 Mean ± SD	T3 Mean ± SD	p*	p**
Energy (kcal)	2626.36 ± 674.44	2419.87 ± 680.44	2576.37 ± 643.53	0.17	–	2649.38 ± 623.36	2576.25 ± 605.56	2514.34 ± 584.44	0.12	–
Carbohydrate (g/day)	354.46 ± 119.25	325.25 ± 105.23	339.23 ± 112.41	0.12	<0.001	372.19 ± 130.19	354.67 ± 127.44	332.40 ± 129.36	0.07	<0.001
Protein (g/day)	84.89 ± 23.19	79.45 ± 19.32	94.26 ± 21.15	0.04	0.003	77.67 ± 18.43	85.71 ± 20.34	96.71 ± 23.62	0.02	0.001
Fat (g/day)	109.41 ± 33.28	91.42 ± 27.83	86.43 ± 23.49	0.001	<0.001	107.57 ± 34.12	98.75 ± 26.43	89.47 ± 25.52	0.03	0.002
Monounsaturated fatty acids (g/day)	35.19 ± 12.44	29.49 ± 11.83	27.38 ± 11.32	0.11	0.01	33.21 ± 13.19	30.14 ± 11.56	28.43 ± 12.13	0.16	0.08
Polyunsaturated fatty acids (g/day)	22.19 ± 10.26	20.65 ± 10.43	18.57 ± 9.36	0.13	0.18	23.29 ± 11.76	20.83 ± 11.43	19.23 ± 10.76	0.19	0.23
Saturated fatty acids (g/day)	35.42 ± 14.26	26.75 ± 10.11	23.32 ± 9.83	<0.001	<0.001	36.59 ± 16.27	33.91 ± 13.73	26.47 ± 10.25	0.003	<0.001
Fiber (g/day)	44.26 ± 19.45	46.19 ± 21.63	51.76 ± 24.27	0.09	0.01	36.15 ± 18.73	45.12 ± 20.63	53.32 ± 23.44	<0.001	<0.001

P*: Calculated by ANOVA, P**: Calculated by ANCOVA after adjusting for energy intake.

There were no significant differences in caloric intake across the LLDS (*p* = 0.17) and HEI (*p* = 0.12) tertiles. Regarding dietary intake, participants in the higher tertiles of LLDS and HEI had significantly higher protein and fiber intake, along with lower fat and saturated fat intake, even after adjusting for energy intake. Additionally, participants in the higher tertiles of LLDS and HEI had significantly lower intake of carbohydrates and monounsaturated fatty acids (MUFA) after adjusting for energy (*p* < 0.05).

Table 3 shows the relationship between LLDS and HEI scores and MIDAS using logistic regression analysis. As shown in Table 3, in the fully adjusted models, higher scores of LLDS and HEI were significantly associated with lower odds of migraine-related disability (OR = 0.68, 95% CI = 0.42–0.96, *p* = 0.02 for LLDS and OR = 0.58, 95% CI = 0.41–0.88, *p* = 0.025 for HEI).

**Table 3:** Multivariate logistic regression analysis examining the effect of LLDS and HEI on migraine-related disability.

Variable		Odds ratio (95% CI)	*p*		*p-trend*
LLDS	Tertile 1		Reference	–	0.02
	Tertile 2		0.52 (0.31–0.76)	0.002	
	Tertile 3		0.45 (0.26–0.69)	0.003	
HEI	Tertile 1		Reference	–	0.03
	Tertile 2		0.57 (0.40–0.79)	0.004	
	Tertile 3		0.49 (0.37–0.72)	0.002	
Model 1	LLDS	Tertile 1	Reference	–	0.02
		Tertile 2	0.56 (0.35–0.79)	0.002	
		Tertile 3	0.57 (0.37–0.81)	0.001	
	HEI	Tertile 1	Reference	–	0.01
		Tertile 2	0.59 (0.42–0.81)	0.005	
		Tertile 3	0.51 (0.38–0.75)	0.002	
Model 2	LLDS	Tertile 1	Reference	–	0.021
		Tertile 2	0.60 (0.38–0.87)	0.004	
		Tertile 3	0.65 (0.43–0.94)	0.023	
	HEI	Tertile 1	Reference	–	0.005
		Tertile 2	0.67 (0.45–0.95)	0.035	
		Tertile 3	0.54 (0.39–0.85)	0.003	
Model 3	LLDS	Tertile 1	Reference	–	0.022
		Tertile 2	0.62 (0.43–0.88)	0.014	
		Tertile 3	0.68 (0.42–0.96)	0.02	
	HEI	Tertile 1	Reference	–	0.003
		Tertile 2	0.70 (0.48–1.09)	0.075	
		Tertile 3	0.58 (0.41–0.88)	0.025	

Model 1: Adjusted for energy intake and BMI; Model 2: Includes Model 1 + gender, Social Status Score, sleep quality and physical activity; Model 3: Includes Model 1 and 2 + water intake, salt consumption and presence of a family history of migraine.

Regarding the relationship between migraine pain intensity and diet quality, Table 4 presents the association between VAS and LLDS and HEI scores. In the fully adjusted model, participants in the highest tertile of LLDS had 45% lower odds of experiencing severe migraine pain compared to those in the lowest tertile (OR = 0.55, 95% CI: 0.38–0.75, *p* = 0.026). Similarly, those in the highest tertile of HEI exhibited a 38% reduction in the odds of severe migraine pain (OR = 0.62, 95% CI: 0.45–0.85, *p* = 0.03).

**Table 4:** Multivariate logistic regression analysis examining the effect of LLDS and HEI on migraine pain intensity.

Variable		Odds ratio (95% CI)		*p-trend*	*p*
LLDS	Tertile 1		Reference	–	0.019
	Tertile 2		0.59 (0.44–0.81)	0.001	
	Tertile 3		0.43 (0.32–0.59)	<0.001	
HEI	Tertile 1		Reference	–	0.026
	Tertile 2		0.62 (0.45–0.83)	0.004	
	Tertile 3		0.45 (0.32–0.63)	0.001	
Model 1	LLDS	Tertile 1	Reference	–	0.015
		Tertile 2	0.63 (0.45–0.84)	<0.001	
		Tertile 3	0.45 (0.33–0.62)	<0.001	
	HEI	Tertile 1	Reference	–	0.018
		Tertile 2	0.66 (0.45–0.89)	0.016	
		Tertile 3	0.48 (0.34–0.69)	0.004	
Model 2	LLDS	Tertile 1	Reference	–	0.026
		Tertile 2	0.66 (0.45–0.89)	0.002	
		Tertile 3	0.49 (0.36–0.68)	0.019	
	HEI	Tertile 1	Reference	–	0.039
		Tertile 2	0.74 (0.53–1.07)	0.12	
		Tertile 3	0.57 (0.36–0.81)	0.023	
Model 3	LLDS	Tertile 1	Reference	–	0.003
		Tertile 2	0.68 (0.46–0.93)	0.017	
		Tertile 3	0.55 (0.38–0.75)	0.026	
	HEI	Tertile 1	Reference	–	0.046
		Tertile 2	0.78 (0.59–1.15)	0.18	
		Tertile 3	0.62 (0.45–0.85)	0.03	

Model 1: Adjusted for energy intake and BMI; Model 2: Includes Model 1 + gender, social status score, sleep quality and physical activity; Model 3: Includes Model 1 and 2 + water intake, salt consumption and presence of a family history of migraine.

Participants in the highest tertile of LLDS and HEI scores exhibited lower odds of migraine-related disability and intensity. These findings suggest an association between diet quality and migraine outcomes, although the cross-sectional design of the study limits the ability to infer causality.

## Discussion

This cross-sectional study found that higher diet quality, as measured by the LLDS and HEI, was associated with reduced severity and frequency of migraine symptoms. However, given the observational nature of the study and the relatively small sample size, these findings should be interpreted with caution. The results highlight a potential link between dietary improvements and migraine management, but they do not establish causality. Future research, including longitudinal studies and clinical trials, is necessary to confirm these associations and explore the underlying mechanisms.

Diet plays a crucial role in influencing health outcomes and the risk of chronic conditions, such as migraines (29, 30). Numerous studies have assessed the impact of individual nutrients or combinations of dietary components on the occurrence and intensity of migraines (13, 31–35). However, few studies have comprehensively assessed overall dietary patterns and their cumulative effects on migraine risk and management. By focusing on dietary quality as a whole, our study provides valuable insights into how a balanced and nutrient-rich diet can mitigate the debilitating effects of migraines.

The HEI-2015 defines high-quality diets as those that emphasize greater intake of nutrient-dense foods, including fruits, vegetables (particularly greens and beans), whole grains, dairy products, total protein sources, seafood, plant-based proteins, and healthy fatty acids (36). It also emphasizes limiting the consumption of sodium, refined grains, added sugars, and saturated fats. Such a diet not only provides essential nutrients required to maintain normal neural function but may also be associated with a reduction in the severity of migraine attacks (37). Similarly, the LLDS is a dietary assessment tool based on food-based principles aligned with the Dutch Dietary Guidelines (27). It evaluates diet quality by assessing adherence to these guidelines, offering insights into how specific dietary patterns may reduce migraine severity and frequency.

Recent studies have consistently shown a significant association between poor diet quality and the prevalence of migraines (29). Supporting our findings, one study demonstrated that healthy women with normal body weight had higher diet quality scores (HEI-2005) compared to those suffering from migraines (38). Similarly, Bakirhan’s research aligns with our results, revealing a negative correlation between total HEI-2015 scores and VAS scores, suggesting that better diet quality is associated with lower migraine severity (39).

Furthermore, another study reported that individuals with migraines tend to consume more pro-inflammatory foods and exhibit lower overall diet quality compared to those without migraines (40). In line with these findings, Ghoreishy et al. observed that individuals with diets high in pro-inflammatory properties had a significantly greater risk of severe headaches compared to those with diets rich in anti-inflammatory foods, which were inversely associated with the frequency and severity of migraine attacks (41).

While the precise mechanisms underlying migraine attacks remain unclear, evidence suggests that inflammation plays a crucial role in their development (42). Additionally, an imbalance between oxidants and antioxidants is believed to contribute to the pathogenesis of migraines, potentially prompting the brain to initiate a homeostatic and neuroprotective response to oxidative stress (37). The association between higher diet quality and reduced migraine symptoms observed in our study may be explained by the presence of antioxidants, unsaturated fatty acids, and dietary fiber in nutrient-dense foods. These components help mitigate oxidative stress and reduce neuroinflammation, potentially preventing or alleviating migraine episodes (39).

From an alternative perspective, it is worth noting that the HEI and LLDS share similarities with dietary patterns such as the DASH (Dietary Approaches to Stop Hypertension) and Mediterranean diets, particularly in their emphasis on fruits, vegetables, and legumes (43, 44). Previous studies have found that greater adherence to the DASH diet is associated with reduced headache severity and shorter headache duration per episode, highlighting the potential benefits of these dietary patterns in managing migraine symptoms (17, 18). Furthermore, studies have shown that the neuroprotective effects of the Mediterranean diet in preventing neurodegeneration are largely attributed to its abundance of bioactive compounds, phytochemicals, and phenolic substances (45–48). These components play a critical role in mitigating inflammation and oxidative stress, which are significant contributors to the development of neurodegenerative conditions and play a key role in the severity of migraine-related disability, as measured by the MIDAS.

As mentioned previously, a possible underlying mechanism for the findings of our study may involve the balance between antioxidants and oxidants, which could be linked to the occurrence of migraines and headaches. Neuroinflammation can result in vasodilation and sensitization of pain-sensitive neurons, primarily through the activation of nociceptors in the trigeminal system. When the trigeminal ganglion is stimulated, it triggers the release of neuropeptides such as substance P, neurokinin, and calcitonin gene-related peptide (CGRP) (49). CGRP plays a critical role in various physiological processes, including the dilation of cerebral and dural blood vessels and the release of inflammatory mediators. Elevated levels of these neuropeptides have been detected in the cerebrospinal fluid of individuals with chronic migraines (50). Additionally, inflammatory markers such as interleukin (IL)-1β, IL-6, and tumor necrosis factor (TNF)-*α* have been observed to increase, particularly during migraine attack phases (51).

Fruits and vegetables, rich in antioxidants, have potential therapeutic effects on migraines due to their bioactive compounds. For instance, indole-3-carbinol and sulforaphane, found in vegetables such as cabbage, broccoli, beets, parsley, spinach, and carrots, may act as CGRP antagonists, demonstrating effectiveness comparable to some medications in certain patients (52). Additionally, diets high in fiber can help reduce inflammation by modulating glucose absorption rates, altering gut microbiota, and decreasing the production of inflammatory cytokines (53). The fermentation of fiber by gut microbiota produces short-chain fatty acids (SCFAs), including butyrate, propionate, and acetoacetate. Among these, butyrate plays a critical role in regulating T-cell function, maintaining gut barrier integrity by enhancing the expression of tight junction proteins, and stabilizing hypoxia-inducible factor (HIF), which supports gut health and reduces toxin permeability (54).

Magnesium also plays a significant role in migraine pathophysiology. Fruits, vegetables, and legumes are excellent sources of magnesium, a mineral often found to be deficient in the plasma and brain tissue of individuals experiencing migraines. Magnesium is essential for mitochondrial energy production and contributes to various physiological processes, including vasoconstriction, inhibition of platelet aggregation, and regulation of serotonin secretion all of which are relevant for managing migraine symptoms (55).

This study is the first to present evidence of an association between the HEI, the LLDS, and migraine-related factors. Dietary data were collected using a validated FFQ, and participants with implausible calorie intakes (>4,000 or < 600 kcal) were excluded from the analysis. To ensure accuracy, interviews were conducted by three trained researchers.

Despite its strengths, this study has several limitations. First, its cross-sectional design precludes establishing causal relationships between diet quality and migraine outcomes. While the findings suggest associations, the temporal relationship between dietary intake and migraine symptoms cannot be determined. Second, the sample size, although sufficient for initial exploratory analyses, was relatively small and drawn from a single clinical setting. This may limit the generalizability of the findings to broader populations, particularly those with different sociodemographic or cultural backgrounds. Third, potential biases, such as recall bias, may have influenced the dietary data collected through the FFQ. Although the FFQ has been validated for the Iranian population, self-reported dietary intake is inherently prone to inaccuracies. Finally, while multivariable logistic regression models were used to adjust for confounders, the number of adjustment variables was limited. This raises the possibility of residual confounding from unmeasured variables, such as other dietary or lifestyle factors, genetic predisposition, or environmental influences. Future studies should aim to include a broader range of confounding factors and employ more robust statistical techniques to minimize bias.

## Conclusion

Based on the findings of our study and the underlying mechanisms, managing overall diet quality, rather than focusing solely on individual macronutrients or micronutrients, appears to be a promising strategy for improving the prognosis and overall condition of individuals with migraines. However, further research is needed to validate the findings of the present study.

## Data Availability

The raw data supporting the conclusions of this article will be made available by the authors, without undue reservation.
